# Effect of short-term room temperature storage on the microbial community in infant fecal samples

**DOI:** 10.1038/srep26648

**Published:** 2016-05-26

**Authors:** Yong Guo, Sheng-Hui Li, Ya-Shu Kuang, Jian-Rong He, Jin-Hua Lu, Bei-Jun Luo, Feng-Ju Jiang, Yao-Zhong Liu, Christopher J. Papasian, Hui-Min Xia, Hong-Wen Deng, Xiu Qiu

**Affiliations:** 1 Division of Birth Cohort Study, Guangzhou Women and Children’s Medical Center, Guangzhou Medical University, Guangzhou 510623, China; 2 Department of Women and Children’s Health, Guangzhou Women and Children’s Medical Center, Guangzhou Medical University, Guangzhou 510623, China; 3 Department of Obstetrics and Gynecology, Guangzhou Women and Children’s Medical Center, Guangzhou Medical University, Guangzhou 510623, China; 4 Center of Bioinformatics and Genomics, Department of Biostatistics and Bioinformatics, Tulane School of Public Health and Tropic Medicine, USA; 5 Department of Basic Medical Science, School of Medicine, University of Missouri – Kansas City, 2411 Holmes St., Kansas City, MO 64108, USA; 6 Department of Neonatal Surgery, Guangzhou Women and Children’s Medical Center, Guangzhou Medical University, Guangzhou 510623, China

## Abstract

Sample storage conditions are important for unbiased analysis of microbial communities in metagenomic studies. Specifically, for infant gut microbiota studies, stool specimens are often exposed to room temperature (RT) conditions prior to analysis. This could lead to variations in structural and quantitative assessment of bacterial communities. To estimate such effects of RT storage, we collected feces from 29 healthy infants (0–3 months) and partitioned each sample into 5 portions to be stored for different lengths of time at RT before freezing at −80 °C. Alpha diversity did not differ between samples with storage time from 0 to 2 hours. The UniFrac distances and microbial composition analysis showed significant differences by testing among individuals, but not by testing between different time points at RT. Changes in the relative abundance of some specific (less common, minor) taxa were still found during storage at room temperature. Our results support previous studies in children and adults, and provided useful information for accurate characterization of infant gut microbiomes. In particular, our study furnished a solid foundation and justification for using fecal samples exposed to RT for less than 2 hours for comparative analyses between various medical conditions.

Shortly after birth, the human intestine is rapidly colonized by numerous microorganisms^1^,^2^. This nascent intestinal microbial community has lasting effects on infant health^3^,^4^,^5^,^6^, including effects on lactate utilization^7^, infection resistance^8^, and development of the immune system^9^,^10^.

Studies based on bacterial 16S ribosomal RNA (rRNA) gene sequencing have revealed the diversity and composition of the infant gut microbiota^11^,^12^. As one of the initial steps that could potentially affect analyses, sample storage conditions are a critical aspect of study design when using DNA-based methods to compare the composition and diversity of microbial communities. It has been generally accepted that different sample types can be stored at freezing temperatures (−80 °C) even for prolonged periods up to several months before DNA extraction, as storage under these conditions does not significantly alter composition of the microbial community^13^,^14^. During sample collection, however, it is not always convenient to analyze fresh samples, or freeze them immediately, leading to exposure of samples to environmental or room temperature (RT) for variable periods before freezing or analysis.

Previous studies have indicated that RT storage might have a considerable impact on the results of analysis conducted with microbial communities of soil^15^, human fecal^16^,^17^,^18^ and sputum samples^19^. In children and adults’ fecal samples, the acceptable time for storage at RT ranged from several hours to a few days. For example, Roesch *et al.*^17^ reported modest (~10%) and gradual changes in the composition and diversity of fecal microbial communities over a period of 12–72 h storage at RT. Cardona *et al.*^18^ and Tedjo *et al.*^20^ observed that RT storage of fecal samples affected the composition of their microbial communities, and concluded that fecal specimens should be frozen immediately at −20 °C, or stored at RT for no more than 24 h prior to analysis.

The gut microbiota of infants is much less diverse and stable than that of adults^2^ and, consequently, it is reasonable to hypothesize that fecal samples of infants, children and adults might be impacted differently by RT storage. Fecal sampling in infants imposes challenges different from children and adults. In common practice, diapers are consistently checked every 0.5–1 hour, and fecal samples collected from the baby’s diapers are promptly (within 1 hour) transferred to the laboratory for further processing (such as storage at −80 °C). Consequently, there is a built in period of storage of infant fecal specimens under non ideal conditions for up to 2 hours prior to freezing or testing. Remarkably, however, studies have not been undertaken to determine whether there are changes in microbial composition of infant fecal samples that are stored for up to 2 hours at RT. We therefore chose to study the effects of up to two hours storage at RT to determine whether this common method of fecal specimen collection from infants might impact the results generated from studies of microbial communities. This is a critical question that needs to be answered in order to validate, or invalidate, this commonly used approach toward large-scale collection of infant fecal samples.

For the current study, we used 16S rRNA pyrosequencing technology to compare bacterial composition of infant fecal samples stored for short periods of time at RT (15 min, 30 min, 1 h and 2 h) to those that were frozen immediately at −80 ^o^C following defecation.

## Methods

### Study population and fecal sample collection

Infant stool samples were obtained from 29 Chinese Han healthy babies (15 neonates aged 1–4 days and 14 infants aged 1–3 months) at the Guangzhou Women and Children’s Medical Center (GWCMC). The subjects were recruited at GWCMC as part of the Born in Guangzhou Cohort Study (BIGCS). Participants with a diagnosis of respiratory infection or digestive tract diseases or a history of treatment with antibiotics or anti-inflammatory agents were excluded. Feces from each subject were collected in sterile flask containers by perianal stimulation. We intentionally collected feces by perianal stimulation, so that we can avoid unknown time period of exposure to temperatures slightly higher than RT in diapers after defecation, and also so that the exposure time to RT is strictly controlled in this study.

To demonstrate the effect of room temparature (at ~25 °C) storage on the microbiota community, each fecal sample was homogenized and partitioned into 5 samples with similar proportions immediately after defecation. One portion was frozen immediately at −80 °C; the remaining four portions were stored at RT for 15 min, 30 min, 1 h or 2 h before freezing in a −80 °C freezer. All samples were stored at −80 °C until further analyses.

### Ethics Statement

This study received approval from the ethics committee of GWCMC, and written informed consent was obtained from parents of each child. The methods were carried out in accordance with the approved guidelines.

### Sample processing and 16S RNA sequencing

Total bacterial DNA was extracted from all samples using a previously reported method^21^. The V3-V5 hypervariable region of the 16S rRNA gene was analyzed to define composition of the bacterial community. Amplification primers were designed with FLX Titanium adapters, primer-F: 5′CCATCTCATCCCTGCGTGTCTCCGACTCAG 3′; primer-R: 5′CCTATCCCCTGTGTGCCTTGGCAGTCTCAG 3′. For amplicon library preparation, 20 ng of each genomic DNA, 1.25U Taq DNA polymerase, 5 μl 10× Ex Taq buffer (Mg^2+^ plus), 10 mM dNTPs (all reagents purchased from TaKaRa Biotechnology Co., Ltd), and 40 pmol primer mix was used per 50 μl amplification reaction. For each sample, the 16S rRNA gene was amplified under the following conditions: initial denaturation at 94 °C for 3 min, followed by 30 cycles of 94 °C for 45 s, 56 °C for 1 min, and 72 °C for 1 min, followed by a final extension at 72 °C for 10 min. PCR products were quantified via gel electrophoresis, pooled and purified for reactions. Pyrosequencing was performed on a 454 GS FLX Titanium sequencer (454 Life Sciences, USA) at BGI-Shenzhen, China.

The raw sequences reported in this article have been deposited in the NCBI BioProject PRJNA312041.

### Data processing and bioinformatics analysis

Low-quality sequences were eliminated from analysis based on the following criteria: a) raw reads shorter than 400 bp; b) a sequence producing more than 8 homopolymers; c) >2 mismatches in the primers, or, d) 1 or more mismatches in the barcode. Pyrosequenced amplicons were removed using the PyroNoise algorithm^22^ in Mothur^23^.

Bioinformatic analysis was implemented using the Quantitative Insights Into Microbial Ecology (QIIME) platform^24^. Briefly, 16S rRNA operational taxonomic units (OTUs) were clustered using an open-reference OTU picking protocol based on 97% nucleotide similarity with the UCLUST algorithm^25^. ChimeraSlayer was employed to remove chimeric sequences^26^. The most abundant sequence from each OTU was selected to determine the phylogeny of the OTU based on taxonomic classifier, RDP-classifier^27^. The relative abundance of each OTU was determined as a proportion of the sum of sequences for each sample. Taxonomic relative abundance profiles (such as, at the phylum, class, order, family and genus levels) were generated based on OTU annotation. To avoid the sampling depth bias, 1,600 reads were randomly selected from each sample when calculating the OTU relative abundance.

The microbial community structure (i.e. species richness, evenness and between-sample diversity) of fecal samples was estimated by biodiversity. Shannon index, phylogenetic diversity, Chao1 index, and the observed number of species were used to evaluate alpha diversity, and the weighted and unweighted UniFrac distances were used to evaluate beta diversity. All of these indices (alpha and beta diversity) were calculated by the QIIME pipeline.

### Statistical analysis

Statistical analysis was implemented using the R platform. Principal coordinate analysis (PCoA) was performed using the “ape” package^28^ based on the UniFrac distances between samples. Principal component analysis (PCA) was performed and visualized using the “ade4” package^29^ based on the relative abundance profiles at phylum, class, order, family and genus levels respectively. In both analyses of the PCoA and PCA, the Wilcoxon rank sum test was used to evaluate the significance of clusters based on the distance between samples on the top two principal components. Permutational multivariate analysis of variance (PERMANOVA) were performed using the “vegan” package^30^, and the permuted *p*-value was obtained by 10,000 times of permutations. Statistical significance was set at *p* < 0.05, and the *q*-value was calculated to evaluate the false discovery rate (FDR) for correction of multiple comparisons.

## Results

### Sequencing coverage

The microbiota composition of all infant fecal samples was characterized by 662,711 filtered high-quality reads, ranging from 1,690 to 9,641 reads per sample (4,909 ± 142 reads/sample; mean ± standard error of mean [SE]). Reads were clustered into 7,458 operational taxonomic units (OTUs) based on 97% nucleotide similarity cutoff for bacterial level phylotypes^31^. 55.2% of these OTUs were robustly classfied into genus-level taxa, and 96.5% could be classified at the family-level. The microbiota of these fecal samples were dominated by Proteobacteria (mean relative abundance 52.3%) and Firmicutes (40.2%), followed by Bacteroidetes (6.1%) and Actinobacteria (0.9%).

### Inter-individual variation accounts for the major difference in fecal samples

Biodiversity analysis was performed to estimate whether short-term RT storage could affect the microbial community structure of fecal samples. In our data set, we assessed the within-sample (alpha) diversity using four estimators: Shannon index, phylogenetic diversity, Chao1 index, and observed number of species. High levels of variation were observed between infants when we examined diversity measures, however, none of these indices increased or decreased significantly with changes in duration of storage at RT (*p* > 0.05 for all comparisons, paired Student’s *t*-test, Fig. S1).

**Figure 1:**
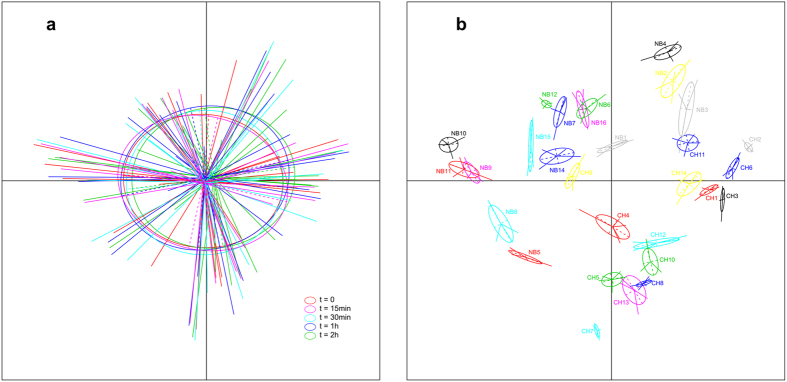
PCoA of the unweighted UniFrac distance as a measure of microbial community structure. (**a**) Samples are grouped by the duration of room temperature storage, showing no clustering of samples in five different durations of storage at room temperature. (**b**) Samples are grouped by individuals, showing apparent separation between these individuals. Samples on the first and second principal coordinates are plotted by nodes. Lines connect samples in the same groups, and colored circles cover the samples near the center of gravity for each group.

The UniFrac distance (both weighted and unweighted) was employed to measure changes in community structure between samples at different RT storage times. Distances were compared using the samples at time 0 (frozen immediately) and samples stored at RT for longer periods. Results of this analysis generated no significant differences between samples across the 15 min–2 h storage time periods and the baseline (immediately frozen) sample (*p* > 0.05 for all comparisons, Student’s *t*-test, Fig. S2). PCoA, which revealed no clustering of samples in the five time points, also failed to detect an effect of duration of storage on overall community structure (*p* > 0.20, Fig. 1a). However, an apparent separation was observed between individuals (*p* < 1e-30, Fig. 1b). These findings suggested that the structural differences of microbial communities in our samples were primarily attributable to inter-individual differences.

The overall picture of the microbial composition of the fecal samples was obtained by PCA, based on the relative abundance profiles of bacterial taxa. For all taxon levels (phylum, class, order, family and genus levels), biplot of the top two principal components showed that these samples were primarily grouped by individuals (*p* < 0.001 at all taxon levels, left figures of Fig. S3), however, no significant division was observed for samples at different time points (*p* > 0.05 at all taxon levels, right figures of Fig. S3). Moreover, applying PERMANOVA at these taxa levels revealed that samples from different individuals were statistically significantly different from each other (*p* < 0.0001 at all taxon levels), but no difference was found from samples between five time points of RT storage (*p* > 0.05 at all taxon levels). These analyses clearly indicated that inter-individual differences, rather than different durations of storage at RT, accounted for the major variations in our total infant samples.

### Compositional change of bacterial taxa during RT storage

**Figure 2:**
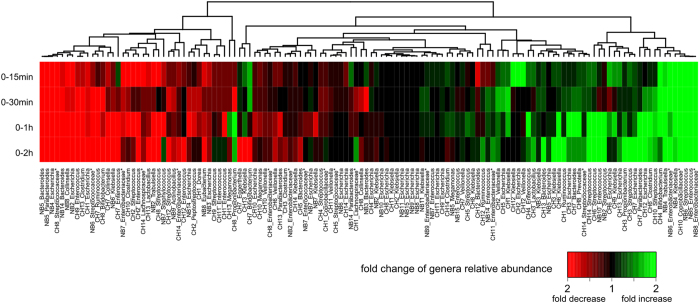
Heatmap representation of the fold change in the relative abundance of genera for all individuals. For each individual, samples stored at room temperature for 15 min, 30 min, 1 h and 2 h are compared with time 0 (frozen immediately). Shades of red and green represent an increase or decrease, respectively, in the relative abundance. Values greater than 2-fold were grouped. To reduce sequencing errors, genera with mean relative abundances <1% at all five time points were removed from analysis. Unclassified genera under a higher taxon (usually at family level) are marked by asterisks. Detailed changes in the relative abundance of genera for all individuals are shown in Fig. S4.

**Figure 3:**
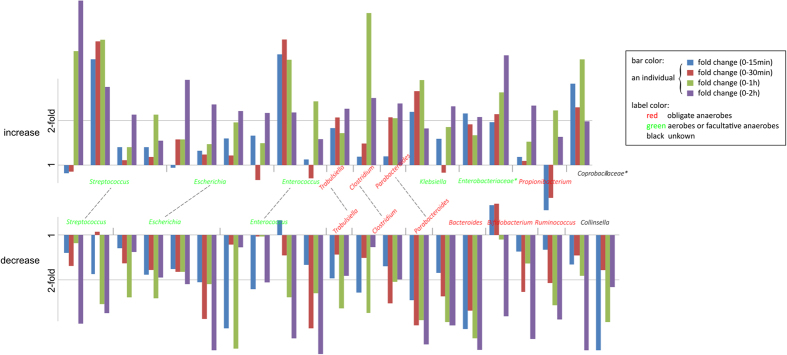
Fold change in the relative abundance of various genera with storage at room temperature. All genera with ≥2 fold increase (top) or decrease (down) after storage in RT for 1 or 2 hours are shown. Genera containing both increased and decreased occurrences were linked with a dotted line. The Y axis represents the fold change in relative abundance, and the X axis reflects different individuals (grouped by four colored bars which indicate the fold change at15 min, 30 min, 1 h and 2 h comparing the baseline). For each individual, genera with mean relative abundances <1% at all five time points were excluded from analysis.

To investigate the change in relative abundance of bacteria due to RT storage, samples at different storage time intervals were compared by the relative abundance profiles for 27 high-abundance genera (mean relative abundance >1% in five samples at 5 different durations of RT storage for each individual). Comparing the samples at time 0 and the samples stored at RT for 15 min, 30 min, 1 h and 2 h, showed that the variations in composition between 0 and 15 or 0 and 30 minute storage intervals was lower than that observed between 0 and 1 or 0 and 2 hour storage intervals (Fig. 2 and Fig. S4, shows the fold change of 130 occurrences of these genera in all individuals, *p* = 0.05, paired Mann-Whitney test). This was largely attributable to the fact that many more genera increased or decreased by more than 2-fold after either 1 or 2 hours storage at RT compared to 15 or 30 minutes storage at RT (Fig. S5). This finding indicated that there were some relatively large fold changes in relative abundance, in individuals, for specific (mainly minor, less abundant) bacterial taxa after storage at RT of 1–2 hours. Due to high inter-individual variability in samples between these infants, however, no genus showed consistent changes in its relative abundance across all individuals (*p* > 0.05 for all genera, paired Mann-Whitney test; Fig. 3, shows changes in the relative abundance of genera that appeared in at least 3 individuals). For most genera, such as the high-occurrence genera *Escherichia*, *Enterococcus* and *Streptococcus*, both increases and decreases in abundance were frequently observed. For *Klebsiella* and the unclassified genera of *Enterobacteriaceae*, however, increases in abundance appeared more frequently than decreases (Fig. 3 and Fig. S4), and decreases in abundance occurred more frequently than increases for some genera of Firmicutes (including *Clostridium*, *Megamonas* and *Ruminococcus*), *Bacteroides* and *Collinsella* (Fig. 3), though these differences were not statistically significant. Additionally, for the 20 occurrences in which the relative abundance of a genus increased by more than 2-fold after 1 h or 2 h at RT, 15 (75%) of these occurrences involved obligate aerobes or facultative anaerobes (Table S1), suggesting that the ability to survive and multiply in the presence of oxygen is an important factor impacting the relative abundance of some microbes in infant fecal samples.

## Discussion

The major conclusion from the current study is that the overall compositional and structural differences of microbial communities in neonatal and infant fecal samples were attributable to inter-individual differences rather than to storage at RT for up to 2 h prior to analysis. PCA analysis at the phylum, class, order, family and genus levels, as well as PCoA analysis at the OTU level showed an obvious separation between individuals, but no significant differences between the five time points of RT storage. These results, with a neonatal and infant population, support the conclusions of previous studies in children and adults^13^,^14^,^32^, which also identified sample separation due to subject rather than to the duration of storage at RT. When using alpha diversity and UniFrac distance to characterize our samples stored at RT for different time periods, we showed that different storage time remained a low-impact factor under the time frame investigated. These findings indicate that microbiota composition and community structure of neonatal and infant fecal samples stored at RT for up to 2 hours is relatively stable and similar to that in samples that are frozen immediately. Given the high level of differences between individuals, and the relatively minor effects of short-term RT storage, comparative analysis across various conditions (such as those contrasting cases and controls for various medical conditions) would be appropriate with samples exposed to RT for up to 2 hours.

Although individual differences accounted for the major variations in composition of microbial communities, changes in the relative abundance of specific taxa were still found during storage at room temperature. Careful examination of Fig. 2, which shows a heatmap representation of the fold change in the relative abundance of genera for all individuals, reveals that the right side of the heat map (representing genera whose relative abundance increase with RT storage), is dominated by facultative anaerobes; obligate anaerobes are a minority. In contrast, examination of the left side of the heatmap, representing genera whose relative abundance decreased with RT storage, reveals that obligate anaerobes dominated. Similarly, analysis of the 20 occurrences in which the relative abundance of a genus increased in an individual by more than 2-fold after 1 h or 2 h storage at RT, revealed that 15 (75%) of these occurrences involved facultative anaerobes, predominantly *Streptococcus, Enterococcus*, and members of the *Enterobacteriaceae*. Facultative anaerobes can survive and replicate in environments containing oxygen, whereas obligate anaerobes may die after relatively brief exposure to oxygen, and cannot replicate in its presence. Consequently, since our samples were exposed to oxygen during RT storage, obligate anaerobes probably did not proliferate, proliferated relatively slowly compared to facultative anaerobes, or died during storage. Thus, although the overall population structure did not change significantly with storage at RT for up to 2 hours, within an individual, there appeared to be a tendency for an increase in the relative abundance of facultative anaerobes compared to obligate anaerobes.

As expected, there were evident differences between the fecal microbiota of neonates and infants found in our study (Fig. S6) vs. previously published studies of adults with regard to both community composition and the magnitude of changes in these communities observed with RT storage^14^. In our samples, the infant fecal microbiota was mainly composed of facultative anaerobes, including *Enterobacteriaceae*, *Streptococcus* and *Enterococcus*, while adult fecal microbiota is generally dominated by obligate anaerobes (e.g. *Bacteroides*, *Prevotella* and *Clostridium spp.*)^4^,^33^. These differences may be attributable to differences in oxygen levels and nutrient availability/composition in infant vs. adult stools/intestines. Differences in bacterial composition of neonatal and infant vs. adult feces could potentially contribute to differential effects of RT storage on the microbial communities in these samples, though our overall findings were comparable to those obtained with adults.

In summary, although we observed changes of specific taxa with increased duration of storage at RT within an individual, our study showed a relatively stable microbiota in infant fecal samples stored at RT for up to 2 hours. The variation of the infant microbiota stored for different lengths of time at RT was relatively small compared to individual differences. However, we recognize that the effects of RT storage may vary with different study designs. In population-based studies, comparative analysis between microbiota of various groups of individuals would not be significantly affected by short-term RT storage. If study of some specific microbial taxa is planned, however, duration of RT storage may be an important factor to consider. In addition, our subjects were healthy infants, and it is possible that low abundance taxa may be affected by RT storage if subjects are ill or treated with antibiotics. Finally, although we studied the impact of RT storage on fecal microbial communities, the actual temperature of fecal specimens collected from a diaper 15 min to 2 hr after defecation, may be somewhat higher.

## Additional Information

**How to cite this article**: Guo, Y. *et al.* Effect of short-term room temperature storage on the microbial community in infant fecal samples. *Sci. Rep.* **6**, 26648; doi: 10.1038/srep26648 (2016).

## Supplementary Material

Supplementary Information
