# Supplementary material for: Effect of short-term room temperature storage on the microbial community in infant fecal samples
Source: Sci Rep. 2016 May 26;6:26648. doi: 10.1038/srep26648 (PMC4880902; doi:10.1038/srep26648)
Supplement: Supplementary Information [file srep26648-s1.doc]

#### Effect of short-term room temperature storage on the microbial community in infant fecal samples

Yong Guo^1,2,†^, Sheng-Hui Li^1,2,†^, Ya-Shu Kuang^1,2,†^, Jian-Rong He^1,2^, Jin-Hua Lu^1,2^, Bei-Jun Luo^3^, Feng-Ju Jiang^3^, Yao-Zhong Liu^4^, Christopher J Papasian^5^, Hui-Min Xia^1,6 *^, Hong-Wen Deng^1, 4^, Xiu Qiu^1,2 *^

^1^ Division of Birth Cohort Study, Guangzhou Women and Children's Medical Center, Guangzhou Medical University, Guangzhou 510623, China

^2^ Department of Women and Children’s Health, Guangzhou Women and Children's Medical Center, Guangzhou Medical University, Guangzhou 510623, China

^3^ Department of Obstetrics and Gynecology, Guangzhou Women and Children's Medical Center, Guangzhou Medical University, Guangzhou 510623, China

^4^ Center of Bioinformatics and Genomics, Department of Biostatistics and Bioinformatics, Tulane School of Public Health and Tropic Medicine, USA

^5^ Department of Basic Medical Science, School of Medicine, University of Missouri – Kansas City, 2411 Holmes St., Kansas City, MO 64108

^6^ Department of Neonatal Surgery, Guangzhou Women and Children's Medical Center, Guangzhou Medical University, Guangzhou 510623, China

^†^ The authors contributed equally to this work.

^*^ Correspondence to:

Xiu Qiu, Division of Birth Cohort Study, Guangzhou Women and Children’s Medical Center, Guangzhou Medical University, 9 Jinsui Road, Guangzhou 510623, China; Phone: 86 2038367162; Fax: 86 2038367162; E-mail: qxiu0161@163.com.

Hui-Min Xia, Division of Birth Cohort Study, Guangzhou Women and Children’s Medical Center, Guangzhou Medical University, 9 Jinsui Road, Guangzhou 510623, China; Phone: 86 2038076019; Fax: 86 2038076019; E-mail: huimin.xia876001@gmail.com.

**Figure S1**: Effect of duration of room temperature storage on alpha diversity indexes, as indicators of biodiversity. Four estimators of the alpha diversity are shown: (**a-b**) Shannon index, (**c-d**) phylogenetic diversity, (**e-f**) Chao1 index, and (**g-h**) observed number of species. For each estimator: values of each individual at five different durations of room temperature storage are plotted by points and connected by solid lines (left figure); alternatively, box plots of the values for all individuals at five different durations of room temperature storage are provided (right figure). None of these indices increased or decreased significantly with changes in duration of storage at room temperature.


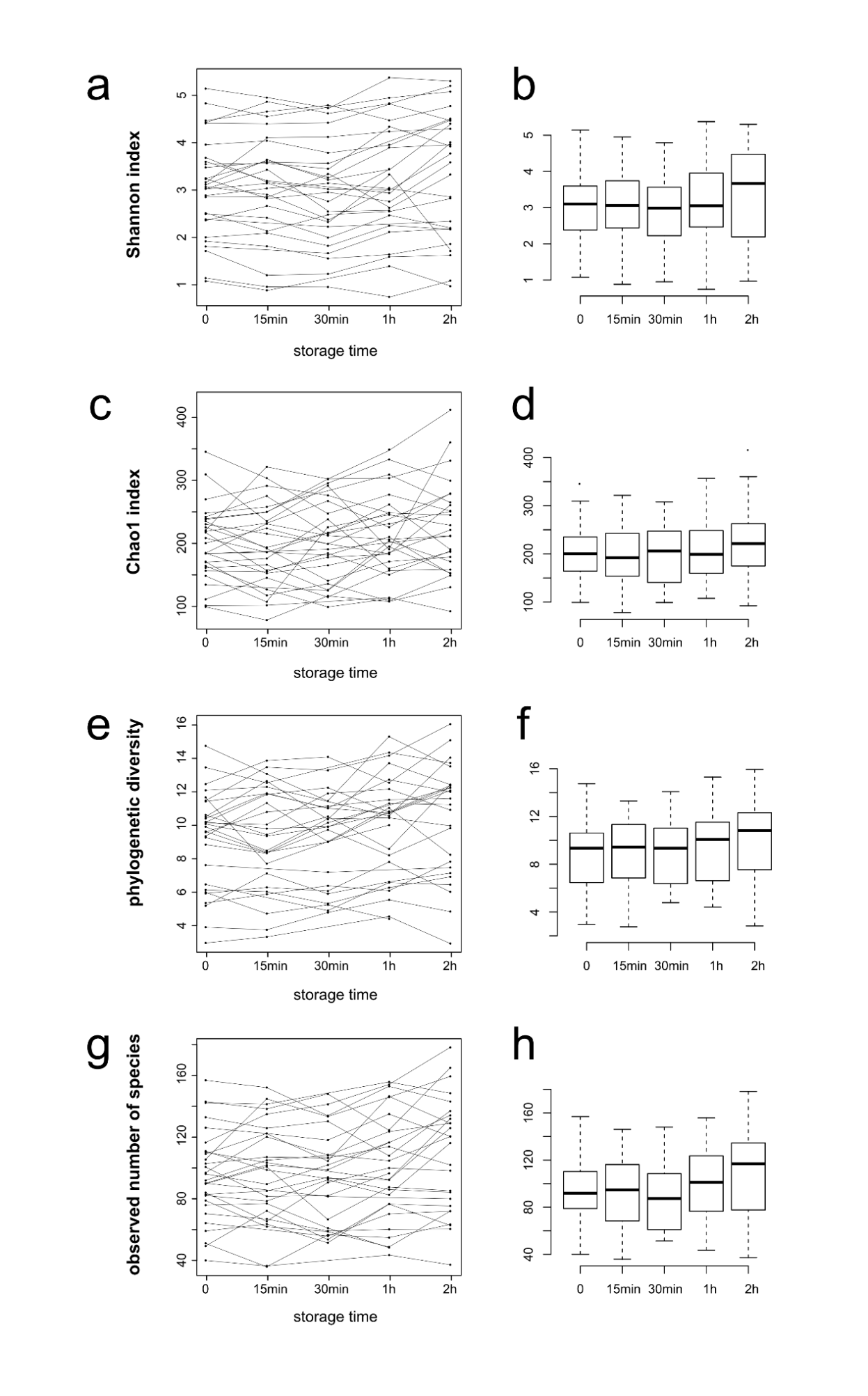


**Figure S2**: Box-and-whisker plots of unweighted (**a**) and weighted (**b**) UniFrac distances as measures of microbial community structure. For each figure, the distances are grouped by samples within the same time points between the baseline (time 0) and other time points (t = 15 min, 30 min, 1 hour and 2 hours). Groups (boxes) were compared by using Student’s t test; for all comparisons, there were no significant differences between these groups.


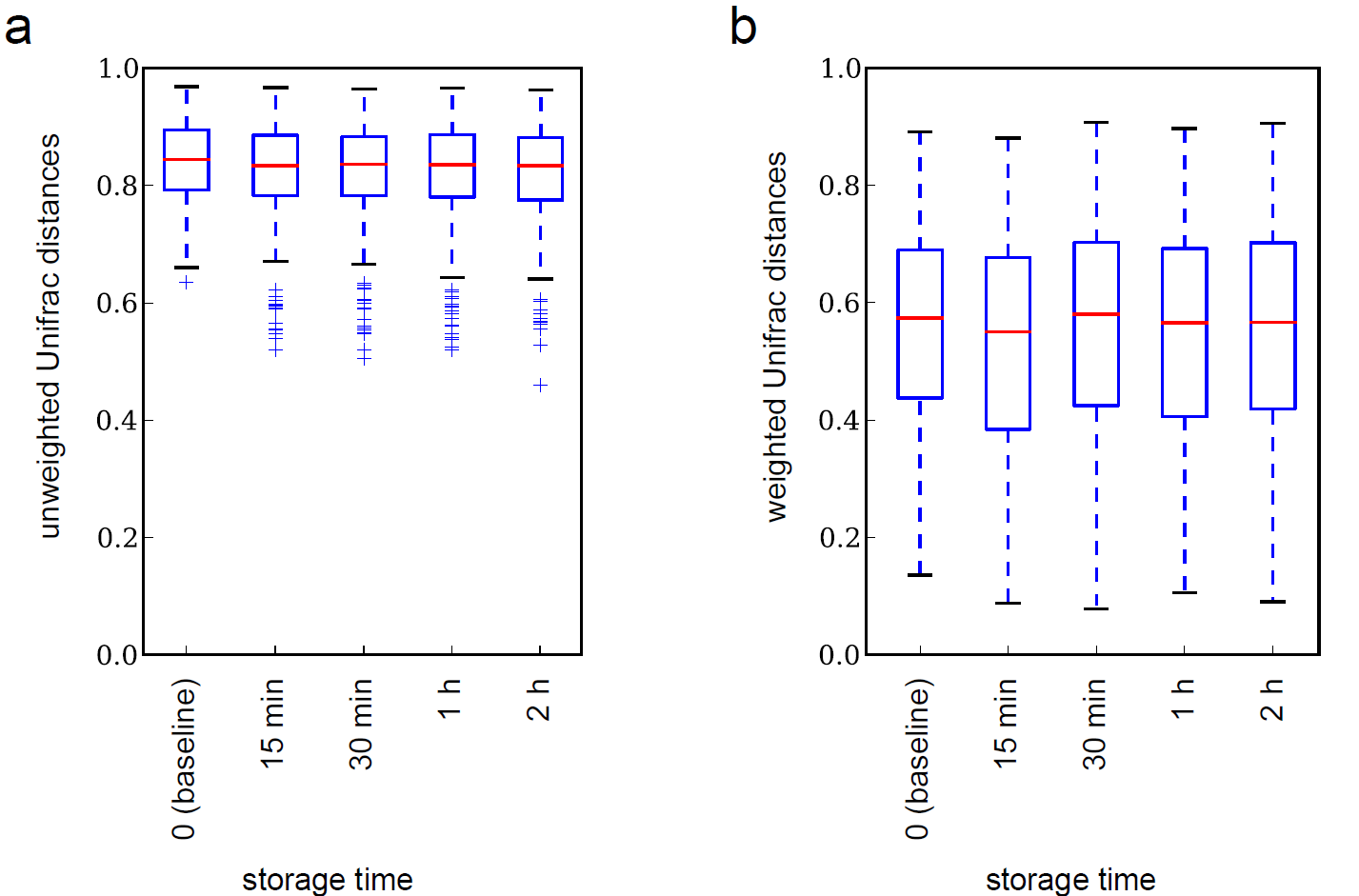


**Figure S3**: PCA analysis at the phylum (**a-b**), class (**c-d**), order (**e-f**), family (**g-h**) and genus (**i-j**) levels. For each taxon level, the left figure is grouped by individuals and the right figure is grouped by samples within the same duration of room temperature storage. Samples on the first and second principal components (PCs) are plotted by nodes. Lines connect samples in the same group, and colored circles cover the samples near the center of gravity for each group. The top six taxa, as the main contributors to microbial composition, are plotted by their loadings in these two PCs. For all taxon levels, these samples are primarily grouped by individuals; no significant division is observed for different durations of room temperature storage.


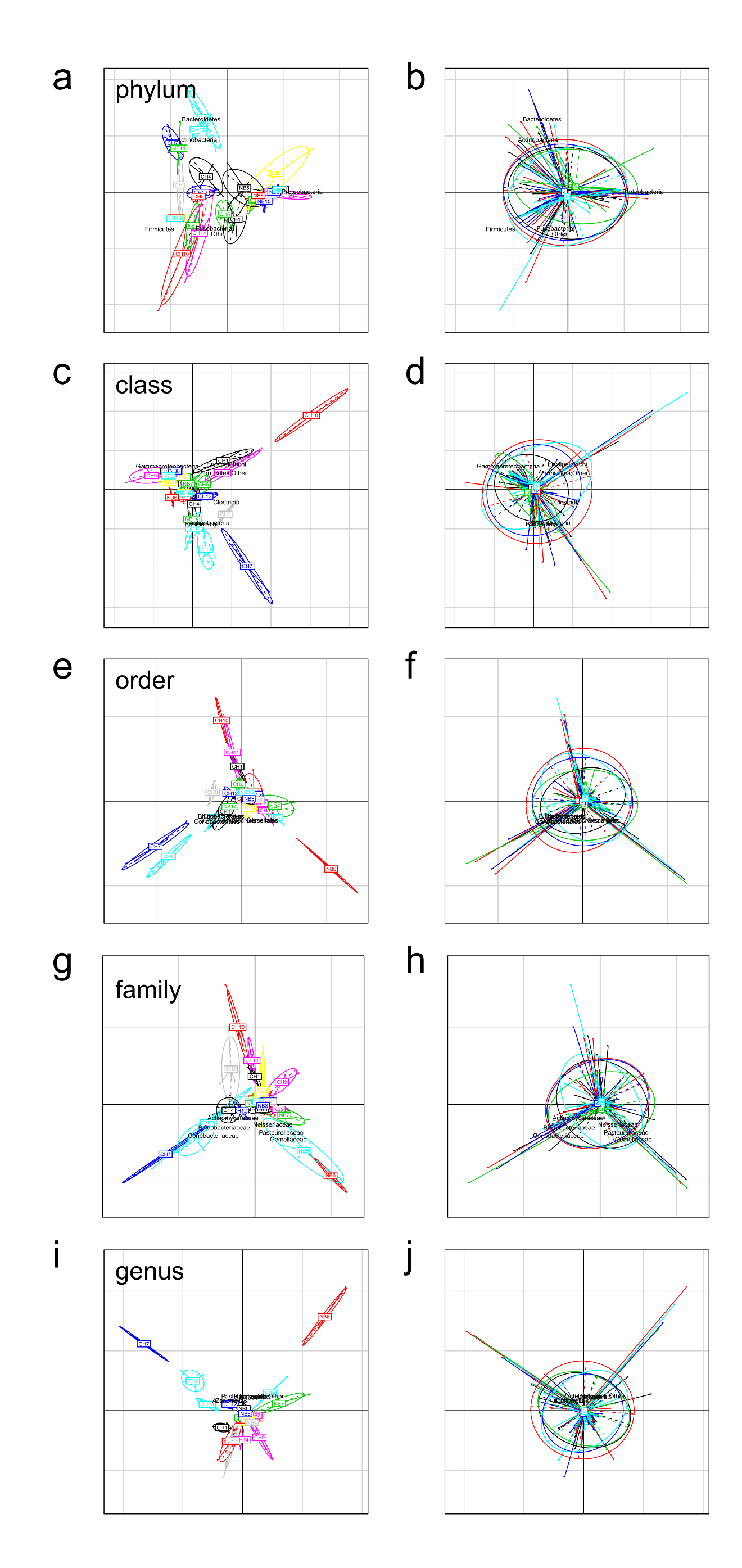


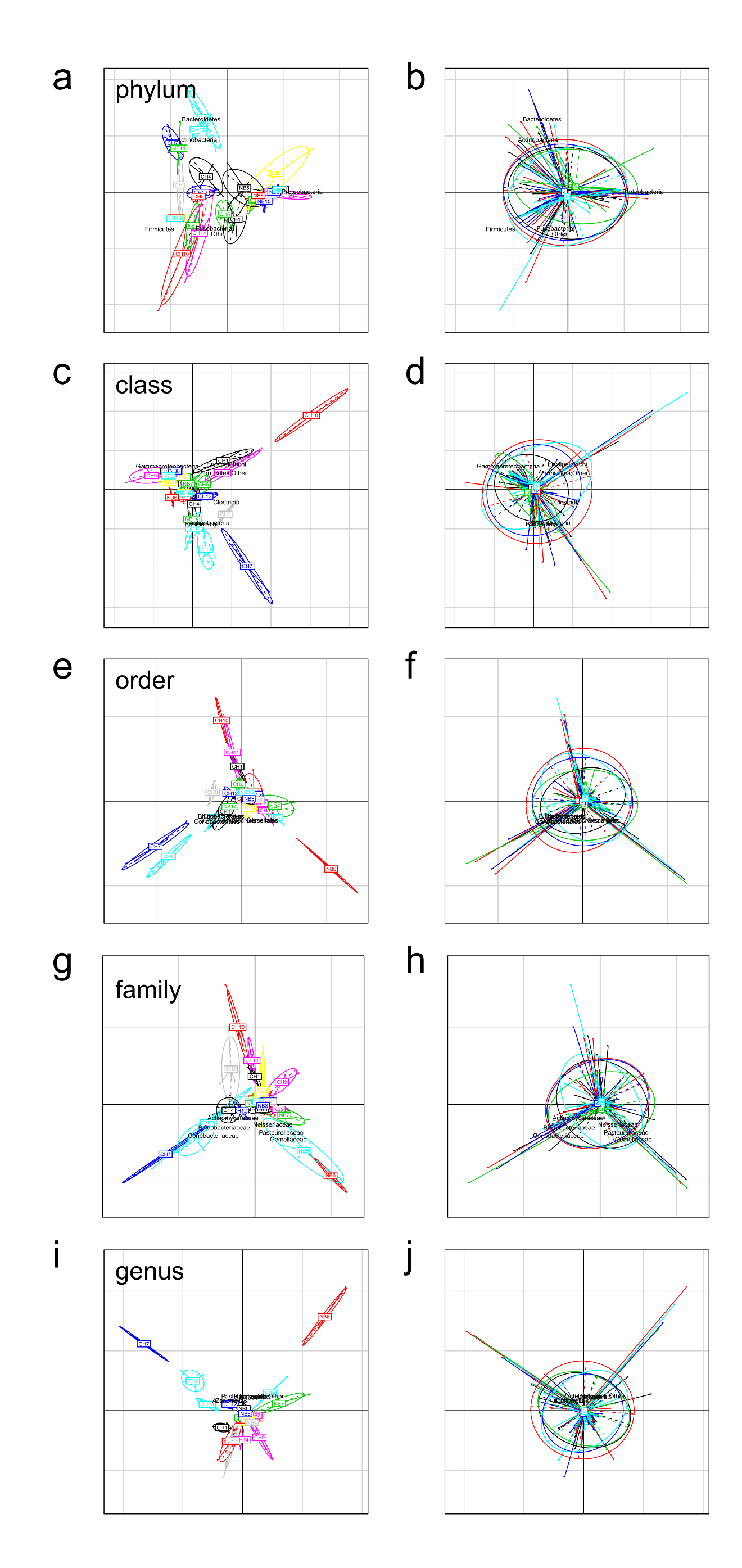


**Figure S4**: Fold change in the relative abundance of genera for all individuals. For each individual, samples stored at room temperature for 15min, 30min, 1h and 2h are compared with the baseline (time 0, frozen immediately). To reduce sequencing errors, genera with mean relative abundance <1% at all five time points were removed from analysis. Fold change is shown on the y axis. Unclassified genera under a higher rank (usually at family level) are marked by asterisks. CH1, CH10, etc, represent samples collected from different individuals. Genera with >2-fold increase or decrease are marked in red.


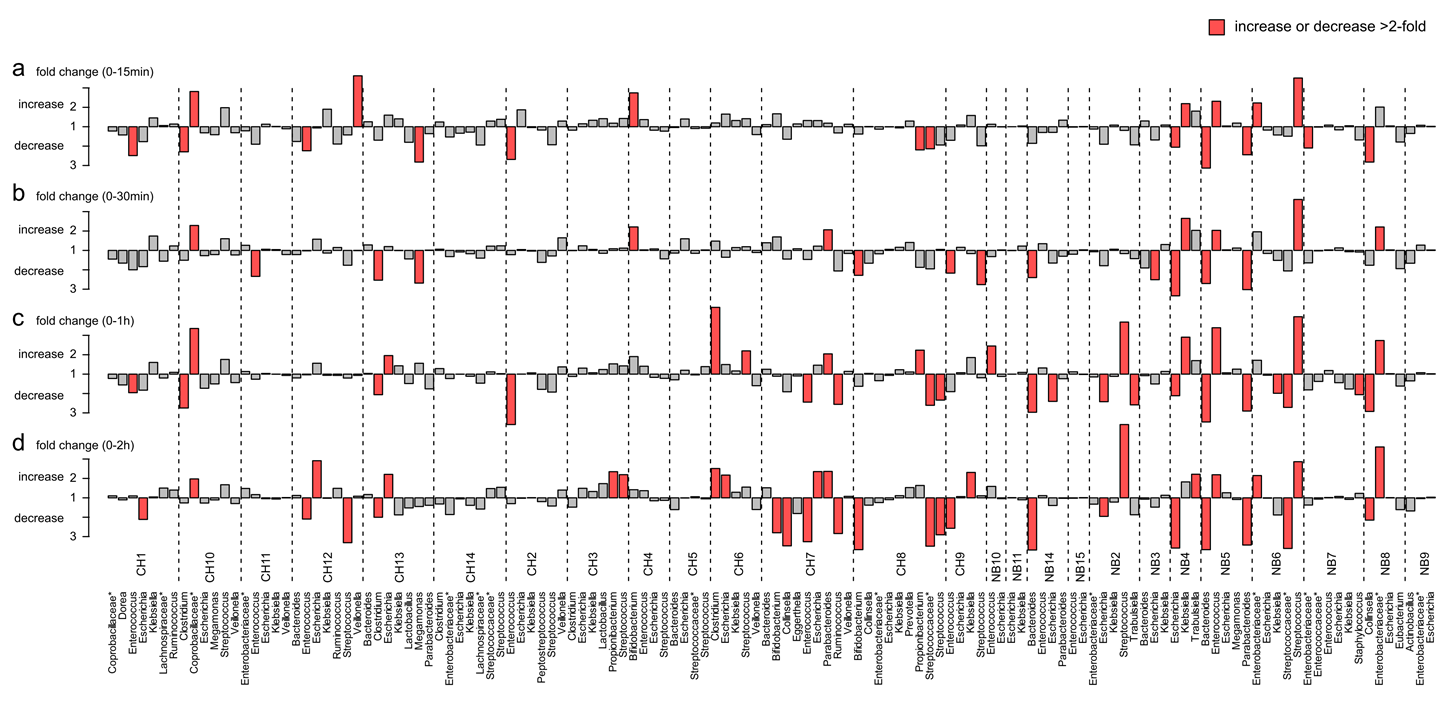


**Figure S5**: Histogram showing the distribution of the fold change in the relative abundance of 27 high abundance genera at different durations of storage at room temperature, 0-15min (**a**), 0-30min (**b**), 0-1h (**c**) and 0-2h (**d**). Values greater than 2 were grouped. The point clearly illustrated in these figures is that the number of genera increasing or decreasing by 2-fold is greater in figures c and d (1 to 2 hours of room temperature storage) than in figures a and b (15 to 30 minutes of room temperature storage). Detailed changes in the relative abundance of genera for all individuals are shown in Figure S4. Genera with >2-fold increase or decrease are marked in red.


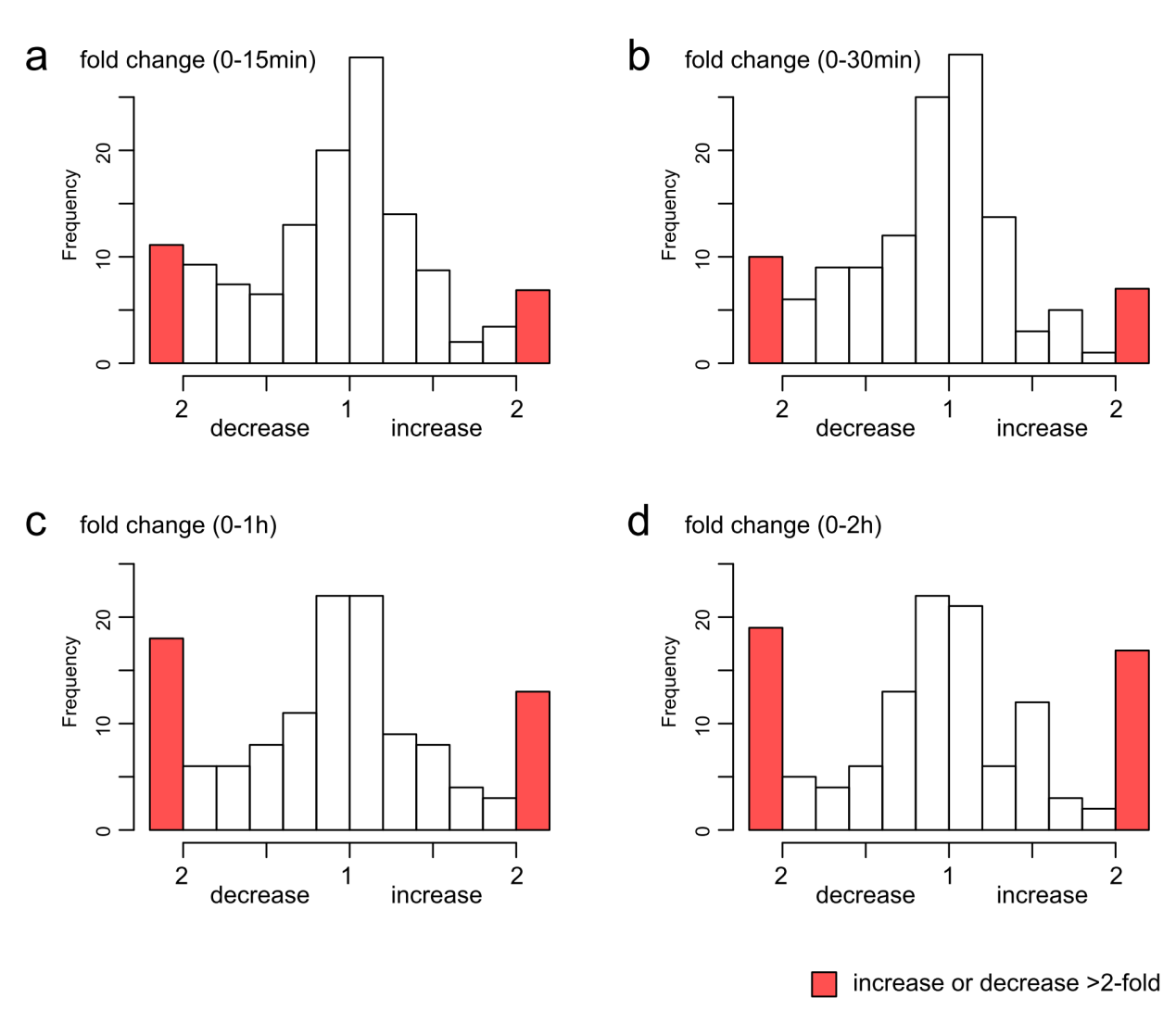


**Figure S6**: The microbial community composition at the phylum level for neonates and infants.


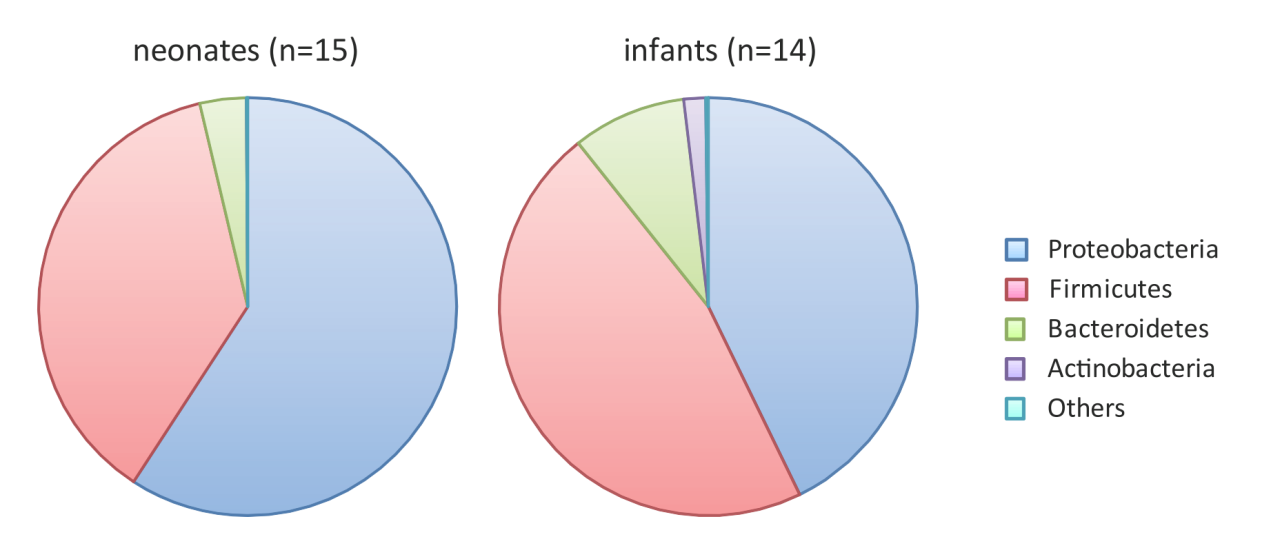


**Table S1**: Taxon associated with 20 occurrences in which the relative abundance of a taxon increased by 2-fold after 1h or 2h at room temperature.

| **Infant** | **Genera** | **Fold change** | | **Oxygen requirement** |
| --- | --- | --- | --- | --- |
|  |  | **0-1h** | **0-2h** |  |
| CH6 | *Clostridium* | 4.4 | 2.5 | Absolute |
| CH10 | *Coprobacillaceae** | 3.4 | 2.0 | Unknown |
| NB6 | *Enterobacteriaceae** | 1.7 | 2.1 | Facultative |
| NB8 | *Enterobacteriaceae** | 2.7 | 3.6 | Facultative |
| NB10 | *Enterococcus* | 2.5 | 1.6 | Facultative |
| NB5 | *Enterococcus* | 3.4 | 2.2 | Facultative |
| CH12 | *Escherichia* | 1.6 | 2.9 | Facultative |
| CH13 | *Escherichia* | 2.0 | 2.2 | Facultative |
| CH6 | *Escherichia* | 1.5 | 2.2 | Facultative |
| CH7 | *Escherichia* | 1.5 | 2.4 | Facultative |
| CH9 | *Klebsiella* | 1.9 | 2.3 | Facultative |
| NB4 | *Klebsiella* | 2.9 | 1.8 | Facultative |
| CH7 | *Parabacteroides* | 2.0 | 2.4 | Absolute |
| CH3 | *Propionibacterium* | 1.5 | 2.3 | Absolute |
| CH8 | *Propionibacterium* | 2.2 | 1.6 | Absolute |
| CH3 | *Streptococcus* | 1.4 | 2.2 | Facultative |
| CH6 | *Streptococcus* | 2.2 | 1.6 | Facultative |
| NB2 | *Streptococcus* | 3.7 | 5.2 | Facultative |
| NB6 | *Streptococcus* | 4.0 | 2.9 | Facultative |
| NB4 | *Trabulsiella* | 1.7 | 2.2 | Facultative |

*: taxonomy assigned at the family level (no classification at the genus level).
